# Allicin Reduces the Production of α-Toxin by *Staphylococcus aureus*

**DOI:** 10.3390/molecules16097958

**Published:** 2011-09-15

**Authors:** Bing-Feng Leng, Jia-Zhang Qiu, Xiao-Han Dai, Jing Dong, Jian-Feng Wang, Ming-Jing Luo, Hong-En Li, Xiao-Di Niu, Yu Zhang, Yong-Xing Ai, Xu-Ming Deng

**Affiliations:** 1 Key Laboratory of Zoonosis, Ministry of Education, Institute of Zoonosis, College of Animal Science and Veterinary Medicine, Jilin University, Changchun 130062, China; Email: llcffnn@gmail.com (B.-F.L.); qiujiazhang1983@163.com (J.-Z.Q.); dai672654190@163.com (X.-H.D.); dongjingletter@163.com (J.D.); wjf927@126.com (J.-F.W.); luomingjing@gmail.com (M.-J.L.); empyreal614@gmail.com (H.-E.L.); drizzlemeng1@yahoo.com (Y.Z.); 2 College of Quartermaster Technology, Jilin University, Changchun 130062, China; Email: niuxd@jlu.edu.cn

**Keywords:** *Staphylococcus aureus*, α-toxin, allicin, sub-inhibitory concentrations

## Abstract

*Staphylococcus aureus *causes a broad range of life-threatening diseases in humans. The pathogenicity of this micro-organism is largely dependent upon its virulence factors. One of the most extensively studied virulence factors is the extracellular protein α-toxin. In this study, we show that allicin, an organosulfur compound, was active against *S. aureus* with MICs ranged from 32 to 64 μg/mL. Haemolysis, Western blot and real-time RT-PCR assays were used to evaluate the effects of allicin on *S. aureus* α-toxin production and on the levels of gene expression, respectively. The results of our study indicated that sub-inhibitory concentrations of allicin decreased the production of α-toxin in both methicillin-sensitive *S. aureus* (MSSA) and methicillin-resistant *S. aureus* (MRSA) in a dose-dependent manner. Furthermore, the transcriptional levels of *agr* (accessory gene regulator) in *S. aureus* were inhibited by allicin. Therefore, allicin may be useful in the treatment of α-toxin-producing *S. aureus *infections*.*

## 1. Introduction

*Staphylococcus aureus* is an important Gram-positive human pathogen that causes myriad diseases, including typical skin and soft tissue infections, and life-threatening invasive diseases such as endocarditis, osteomelytis, pneumonia and toxinosis [1]. Furthermore, it is also a significant pathogen that is responsible for intramammary infection in dairy cattle, sheep and goats [2]. Due to the wide spread of methicillin-resistant *S. aureus* (MRSA), the morbility and mortality of *S. aureus *infections remain high in spite of antimicrobial chemotherapy [3]. Consequently, the severity of the diseases caused by this organism has heightened the urgent need for alternative antimicrobial classes or therapeutic strategies.

*S. aureus* secretes a number of virulence factors that contribute to its pathopoiesis. α-Toxin is one of the major exotoxins produced by most *S. aureus* strains during the post-exponential to stationary growth phase [4]. α-Toxin is secreted as a 33.2 kDa soluble polypeptide which possesses numerous biological functions. The effects of α-toxin are both concentration- and cell type-dependent and include cell lysis [5], release of proinflammatory mediators and cytokines [6], and induction of apoptosis [7]. It has been well manifested that α-toxin plays critical role in many *S. aureus* infections, such as intraperitoneal, intramammary, and corneal infection, as well as staphylococcal pneumonia, as strains lacking α-toxin are less virulent in animal models of diseases [8,9,10,11]. 

Recently, anti-virulence approach as an alternative strategy for the treatment of bacterial infections has gained increased interest [12]. Considering the role of α-toxin in disease, it could be an important target for the development of anti-virulence agents to combat *S. aureus*-mediated diseases [9]. Such strategy relies on newly discovered synthetic or natural small organic compounds that possess anti-virulence activity [12]. It has been reported that the production of α-toxin in *S. aureus* could be affected by some natural compounds [13,14]. Allicin is one of the active principles of freshly crushed garlic homogenates, has various biological properties, including antibacterial, antifungal, antiparasitic, antiviral, anti-inflammatory and immunomodulatory activities [15,16,17,18,19]. In this study, we are aimed to investigate the anti-*S. aureus *activity of allicin, and further determine the influence of sub-inhibitory concentrations of allicin on the expression of α-toxin via haemolysis, western-blot and real-time RT-PCR assays.

## 2. Results

### 2.1. Influence of Magnolol on S. aureus Growth

The MICs of oxacillin (one of the β-lactam antibiotics) and allicin against *S. aureus* strains are shown in Table 1. Allicin was active against MSSA and MRSA strains. The MIC values of allicin against four strains producing α-toxin were 64 μg/mL. These data were in accordance with previous study [20]. The MICs of allicin show no remarkable difference between MSSA and MRSA strains. Our result indicated that the structure of allicin could be used as a basic structure for designing novel and more potent drugs to treat against *S. aureus*.

**Table 1:** Bacterial strains used in the study and their MICs to allicin.

S. aureus strains	Description	Source	MIC (μg/mL)	
			Oxacillin	Allicin
ATCC 29213	β-Lactamase-producing oxacillin-susceptible strain, α-toxin-producing strain	ATCC	0.25	64
ATCC 10832	Wood 46, a natural isolate that produces high levels of α-toxin	ATCC	0.125	64
BAA-1717	USA300-HOU-MR, Isolated from adolescent patient with severe sepsis syndrome in Texas Children's Hospital, α-toxin-producing strain	ATCC	256	64
8325-4	A high-level α-toxin-producing strain derived from NCTC 8325	Timothy J. Foster	0.125	64
DU 1090	α-toxin-negative mutant of *S. aureus* 8325-4,prepared by insertion of a transposon in the *hla* gene	Timothy J. Foster	0.125	32
ATCC 25923	A clinical isolate collected at Seattle in 1945	ATCC	0.25	32

**Figure 1:**
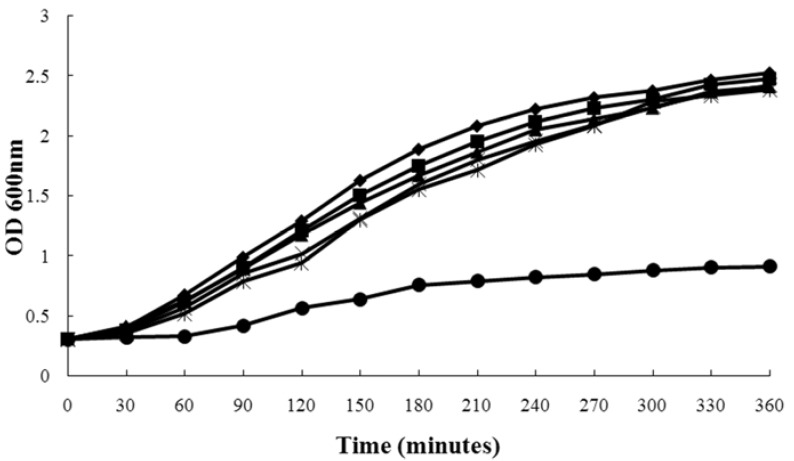
Growth curves of *S. aureus* strain ATCC 29213 treated with different concentrations of allicin. (◆), untreated *S. aureus*; (■), *S. aureus *plus allicin at 2 μg/mL; (▲), *S. aureus *plus allicin at 4 μg/mL; (×), *S. aureus* plus allicin at 8 μg/mL; and (*), *S. aureus *plus allicin at 16 μg/mL; (●), *S. aureus *plus allicin at 64 μg/mL.

Figure 1 shows the growth curve of *S. aureus* strain ATCC 29213 grown in the presence of increasing concentrations of allicin, wherein we found that 2-16 μg/mL of allicin had little influence on *S. aureus* growth, while at 64 μg/mL, the growth of *S. aureus* was markedly inhibited. Furthermore, the growth of *S. aureus* strains ATCC 10832, BAA-1717 and 8325-4 was not affected by these concentrations of allicin in the same way. In summary, the addition of 2-16 μg/mL of allicin displayed little influence on these strains’ growth (data not shown).

### 2.2. Allicin Reduces α-Toxin Levels in S. aureus Culture Supernatants

Four α-toxin-producing *S. aureus* strains were exposed to graded sub-inhibitory concentrations of allicin to the post-exponential phase. The haemolytic activities of the culture supernatants are shown in Table 2. When grown in the presence of 2 μg/mL of allicin, the haemolysis values of 8325-4, ATCC 29213, 10832 and BAA-1717 culture supernatants were 96.5, 89.2, 64.1 and 90.7%, respectively, compared with a allicin-free culture. Notably, supplementation with 16 μg/mL of allicin led to substantial inhibition of the haemolysis of *S. aureus* strains 8325-4, ATCC 29213, 10832 and BAA-1717. As expected, a dose-dependent attenuation of haemolysis was observed in all tested strains. 

**Table 2:** Haemolysis of *S. aureus* culture supernatants treated with increasing concentrations of allicin.

	Haemolysis (%) of rabbit erythrocytes by culture supernatant ^a^				
Strains	0	2 μg/mL	4 μg/mL	8 μg/mL	16 μg/mL
8325-4	100	96.5 ± 3.0	75.1 ± 4.9 *	64.3 ± 4.1 *	22.1 ± 4.0 *
ATCC 29213	100	89.2 ± 4.5	62.5 ± 4.4	36.5 ± 5.1 *	15.6 ± 3.5 **
ATCC 10832	100	64.1 ± 4.7	43.2 ± 3.7 *	30.0 ± 3.9 *	NO
BAA-1717	100	90.7 ± 5.2	58.1 ± 4.4 *	24.3 ± 4.7 *	6.23 ± 3.2 **

^a^ The culture supernatants without allicin served as the 100% haemolysis control. NO represents that there was no observed haemolytic activity. Values represent the mean and standard deviation of three independent experiments. * indicates *p* < 0.05 and ** indicates *p* < 0.01, when compared with the corresponding control.

**Figure 2:**
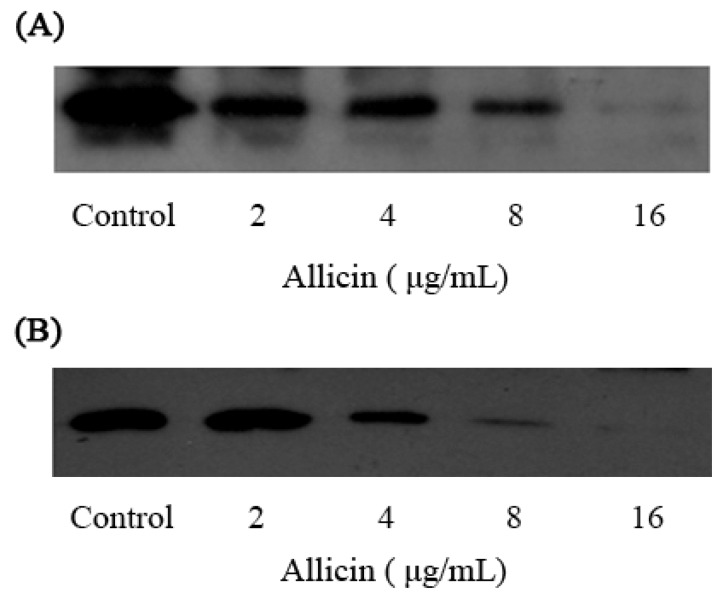
Western blot analysis of α-toxin production by strain ATCC 29213 (**A**) and BAA-1717 (**B**) after treatment with different concentrations of allicin.

**Figure 3:**
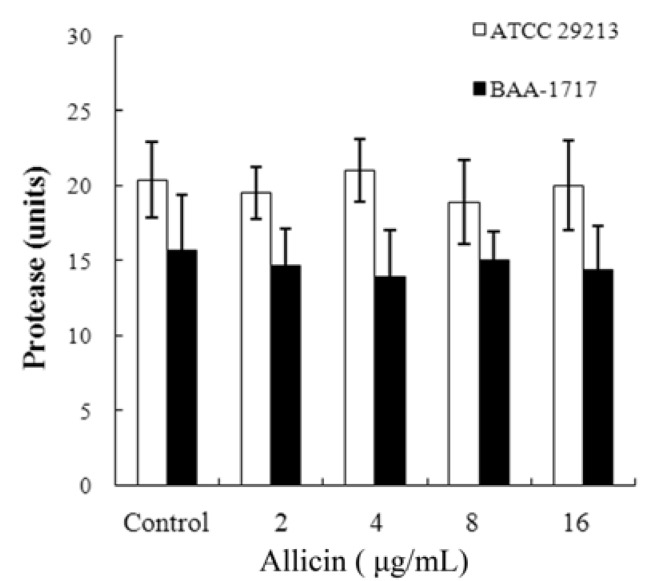
Protease units of *S. aureus *culture supernatants after treatment with allicin. Values represent the mean ± SD for three independent experiments.

Western blot analysis indicated that allicin decreased in a dose-dependent manner the production of α-toxin by *S. aureus* (Figure 2). Exposure to 2 μg/mL of allicin may lead to a visible reduction in α-toxin production, and none or little protein could be detected in all tested strains while at 16 μg/mL. In addition, growth in the presence of increasing concentrations of allicin did not influence protease secretion (Figure 3). Therefore, the decrease in α-toxin production by *S. aureus* was not owing to an increase in protease secretion induced by allicin.

### 2.3. Allicin Inhibits the Transcription of hla and agrA in S. aureus

**Figure 4:**
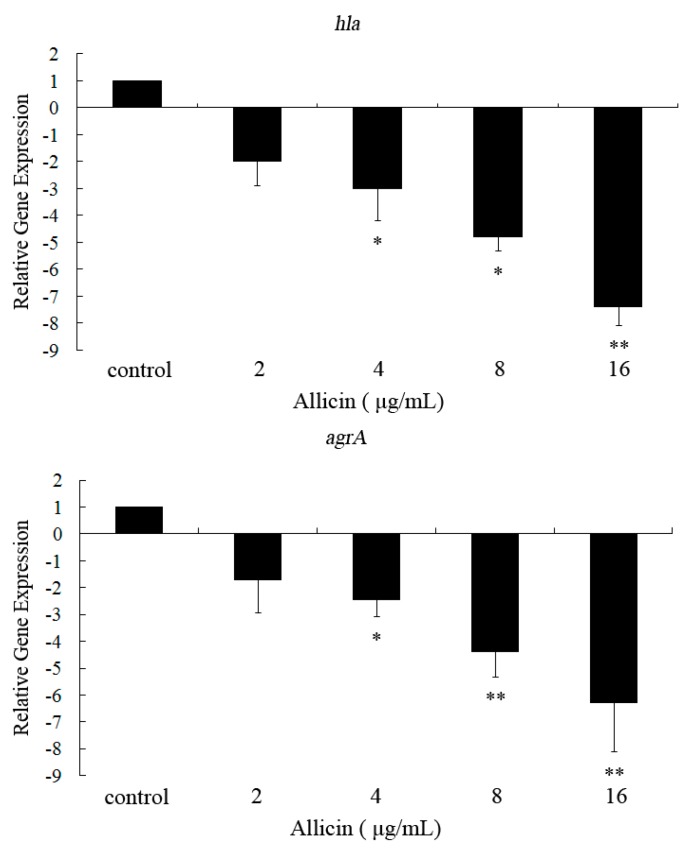
Effectsof different concentrations of allicin on the transcription of *hla* and *agrA* in *S. aureus*. Data are expressed as the means ± SD for three independent experiments. The Student’s t-test was used to determine statistical differences. * Indicates *p* < 0.05 and ** indicates *p* < 0.01 *vs*. control.

Figure 4 shows the transcriptional levels of *hla* and *agrA* in *S. aureus* ATCC 29213 after treatment with graded sub-inhibitory concentrations of allicin. When exposed to 16 μg/mL of allicin, the transcriptional levels of *hla* and *agrA* were reduced by 7.4- and 6.3-fold, respectively. Both genes were affected by sub-inhibitory allicin in a dose-dependent fashion.

## 3. Discussion

For many years, with the abuse of traditional antimicrobials and the decrease in development of new antibacterial agents, increasing numbers of *S. aureus* strains have become MSRA strains, which are spread in communities, leading to dramatic changes in epidemiology and disease incidence. Numerous virulence factors produced by *S. aureus* play a significant role in the pathogenesis of infection (e.g., α-toxin, enterotoxins, toxic shock syndrome toxin 1, and cell wall-associated proteins). Therefore, the clinical efficacy of new antibacterial agents used for the treatment of *S. aureus* infections should depend on the respective bacteriostatic or bactericidal effects and the ability to prevent virulence factor produced by bacteria. For the management of toxic *S. aureus* infections, some antibiotics display an anti-virulence activity at concentrations below the MIC. For instance, protein synthesis-suppressing antibiotics, such as clindamycin and linezolid, are recommended for the treatment of *S. aureus*-produced toxic syndromes, as concentrations below the MIC have been shown to impair the expression of *S. aureus *virulence factors [21,22]. On the contrary, β-lactam antibiotics have proven to be unfavourable because even sub-inhibitory concentrations (for example, of methicillin) lead to an increase in α-toxin expression through a stimulatory effect on exoprotein synthesis [23]. 

Allicin is the active compound of garlic, an edible plant which has generated a lot of interest throughout human history as a medicinal panacea. It has been shown to exhibit a wide spectrum of antibacterial activity against Gram-negative and Gram-positive bacteria, including species of *Escherichia*, *Salmonella*, *Staphylococcus*, *Streptococcus*, *Klebsiella*, *Proteus*, *Bacillus*, and *Clostridium *[20]. The main mechanism involved in the antimicrobial effect was due to the inhibition of certain thiol-containing enzymes in the microorganisms by the rapid reaction of thiosulfinates with thiol groups [24]. In addition to its antimicrobial activity, allicin also exhibits anti-virulence property. Gonzalez-Fandos *et al*. reported that allicin could prevent the formation of staphylococcal enterotoxins A, B, and C1 and thermonuclease [25]. More recent studies by Arzanlou *et al*. have shown that low concentrations of allicin could inhibit the haemolytic activities of pneumolysin O (PLY) and streptolysin O (SLO) [26,27]. They also indicated that allicin inhibits PLY and SLO by binding to cysteinyl residue in the binding site [26,27]. In the present study, we demonstrate that sub-inhibitory concentrations of allicin could dose-dependently reduce the haemolytic activity in *S. aureus* via inhibiting the production of α-toxin. Unlike PLY and SLO, there are no cysteines in the structure of α-toxin [4]. Consequently, we may preclude the binding of allicin with cysteinyl residue. Based on the views that α-toxin plays significant roles in the pathopoiesis of *S. aureus* infections [15,16,17,18,19], α-toxin may be potentially served as an important target for the development of anti-virulence chemotherapy, and our data indicate that the allicin structure may be used as a basic structure for the development of novel anti-infective drugs that aims to *S. aureus* α-toxin. 

The production of α-toxin in *S. aureus* was controlled by a number of regulators, such as Agr, Sae, Sar, and Rot [28]. The transcriptional levels of *agrA*, which postitively regulates the expression of *hla* [29], was significantly inhibited in *S. aureus* after treatment with allicin. Consequently, the mode of action that allicin reduces the production of α-toxin may, in part, due to the inhibition of Agr regulatory system.

## 4. Experimental

### 4.1. Bacterial Strains and Reagents

Allicin was purchased from the National Institute for the Control of Pharmaceutical and Biological Products (Beijing, China), The bacterial strains used in the study are listed in Table 1. *S. aureus* strains ATCC 29213, 10832, BAA- 1717 and 8325-4, which have the potency to produce α-toxin, were used to investigate the effect of allicin on *S. aureus* α-toxin production, and stock solutions of various concentrations were prepared in dimethyl sulfoxide (DMSO) (Sigma-Aldrich, St Louis, MO, USA).

### 4.2. MIC Determination

The minimal inhibitory concentrations (MICs) of allicin for *S. aureus* were determined using the broth microdilution method in Mueller–Hinton broth (MHB) (BD Biosciences, Sparks, MD, USA) according to CLSI guidelines [30]. MIC values were defined as the lowest drug concentration where no bacterial growth was observed. 

### 4.3. Growth Curves

Bacteria were cultivated at 37 °C to an OD value of 0.3 at 600 nm in MHB, and 100 mL volumes of the precultures were transferred into six 250-mL Erlenmeyer flasks, followed by the addition of allicin at concentrations of 2, 4, 8 and 16 μg/mL. The final DMSO concentration for all cultures was 1% (v/v). The control culture contained 1% DMSO only. Following the addition of allicin (or DMSO), bacteria were further cultured at 37 °C with constant shaking under aerobic conditions. The growth of cells was monitored by reading the OD_600 nm_ values at 30 min intervals.

### 4.4. Haemolysis Assay

*S. aureus* strains were grown in MHB in the absence or presence of graded sub-inhibitory concentrations of allicin until reaching the post-exponential growth phase (OD_600 nm_ of 2.5, 2.0, 2.0 and 2.5 for strains ATCC 29213, 10832, BAA- 1717 and 8325-4, respectively). Bacterial supernatants were collected by centrifugation (5,500 × g, 4 °C, 1 min), the supernatant was collected, and the residual cells were removed using a 0.2 μm filter. Prior to the addition of 25 μL of defibrinated rabbit blood, a 0.1 mL volume of culture supernatant was brought up to a volume of 1 mL through the addition of PBS buffer. After incubation for 15 min at 37 °C, the unlysed blood cells were pelleted by centrifugation (5, 500 × g, room temperature, 1 min). The hemolytic activity of the supernatant was detected by measuring the optical density at 543 nm. The control culture supernatant served as the 100% hemolysis control, and the percent hemolysis was calculated by comparison with the control culture.

### 4.5. Western Blot Analysis and Proteolytic Activity Assay

The culture supernatants described earlier were also employed for Western blot analysis. An equal volume (25 μL) of culture supernatant was loaded onto a 12% sodium dodecyl sulfate-polyacrylamide gel after boiling in Laemmli sample buffer [31]. Protein was transferred onto polyvinylidene fluoride membranes (Wako Pure Chemical Industries, Ltd, Osaka, Japan). The membranes were blocked for 2 h using 5% bovine serum albumin (Wako) in PBS. Antibody to α-toxin was purchased from Sigma-Aldrich and diluted to 1:8000; then a horseradish peroxidase-conjugated anti-rabbit antiserum (Sigma-Aldrich), diluted 1:4000, was used as the secondary antibody. The blots were developed using Amersham ECL Western blotting detection reagents (GE Healthcare, Buckinghamshire, UK). 

### 4.6. RNA Isolation and Real-Time RT–PCR

*S. aureus* ATCC 29213 was cultivated in MHB with or without graded sub-inhibitory concentrations of allicin to the post-exponential growth phase (OD_600 nm_ of 2.5), and total RNA was prepared based on our previously described method [32]. Cells were harvested by centrifugation (5,000 × g for 5 min at 4 °C) and resuspended into TES buffer (10 mM Tris-Cl, 1 mM EDTA, 0.5% SDS) containing 100 μg/mL of lysostaphin (Sigma-Aldrich). Following incubation at 37 °C for 10 min, a Qiagen RNeasy Maxi column was used to isolate total bacterial RNA, which was in accordance with the manufacturer’s instructions. The contaminating DNA was removed using the optional on-column RNase-free DNase I step (Qiagen, Hilden, Germany). RNA concentrations were detected from the OD_260_ nm, and the RNA was loaded onto an RNase-free 2% agarose gel to test for generalized degradation. The primer pairs used in real-time RT-PCR are listed in Table 3. RNA was reverse transcribed into cDNA using the Takara RNA PCR kit (AMV) Ver. 3.0 (Takara, Kyoto, Japan), according to the manufacturers’ protocol. The resulting cDNA was stored at −20 °C until it was required. The PCR reactions were carried out in a 25 μL volume and contained SYBR Premix Ex TaqTM (Takara), as recommended by the manufacturer. The reactions were performed using the 7000 Sequence Detection System (Applied Biosystems, Courtaboeuf, France). Cycling conditions were as follows: 95 °C for 30 s; 30 cycles at 95 °C for 5 s, 55 °C for 30 s, and 72 °C for 40 s; and one dissociation step of 95 °C for 15 s, 60 °C for 30 s, and 95 °C for 15 s. All samples were analyzed in triplicate, and the *16S* *rRNA * housekeeping gene served as an internal control to normalize the expressional levels between samples. The relative expression levels were analyzed by the △△Ct method that is described in the Applied Biosystems User Bulletin No. 2.

**Table 3:** Primers used in real-time RT-PCR.

Primer	Sequence	Location within gene
*16S rRNA-fw*	5'-GCTGCCCTTTGTATTGTC-3'	287–305
*16S rRNA-rv*	5'-AGATGTTGGGTTAAGTCCC-3'	446–465
*hla-fw*	5'-TTGGTGCAAATGTTTC-3'	485–501
*hla-rv*	5'-TCACTTTCCAGCCTACT-3'	569–586
*agrA-fw*	5'-TGATAATCCTTATGAGGTGCTT-3'	111–133
*agrA-rv*	5'-CACTGTGACTCGTAACGAAAA-3'	253–274

### 4.7. Statistical Analysis

SPSS 12.0 statistical software was applied to analyze the experimental data. The data are presented as the mean value ± SD. An independent Student’s t-test was used to determine statistical differences, and a *p *value less than 0.05 was considered to be statistically significant.

## 5. Conclusions

In this study, we have investigated the influence of allicin on the secretion of α-toxin by *S. aureus*. Our date showed that sub-inhibitory concentrations of allicin inhibited the production of α-toxin in both MSSA and MRSA in a dose-dependent way. Therefore, these findings indicate that allicin may be useful in the treatment of infections with α-toxin-producing *S. aureus.*
